# Atorvastatin inhibits pro-inflammatory actions of aldosterone in vascular smooth muscle cells by reducing oxidative stress

**DOI:** 10.1016/j.lfs.2019.01.043

**Published:** 2019-02-02

**Authors:** Thiago Bruder-Nascimento, Glaucia E. Callera, Augusto C. Montezano, Eric J. Belin de Chantemele, Rita C. Tostes, Rhian M. Touyz

**Affiliations:** a Department of Pharmacology, Ribeirao Preto Medical School, University of Sao Paulo, Brazil; b Kidney Research Centre, University of Ottawa, Canada; c Vascular Biology Center, Medical College of Georgia, Augusta University, United States of America; d Institute of Cardiovascular and Medical Sciences, University of Glasgow, UK

**Keywords:** Inflammation, Vascular, Statins, Mineralocorticoids, Redox signaling

## Abstract

Vascular inflammatory responses play an important role in several cardiovascular diseases. Of the many pro-inflammatory vasoactive factors implicated in this process, is aldosterone, an important mediator of vascular oxidative stress. Statins, such as atorvastatin, are cholesterol-lowering drugs that have pleiotropic actions, including anti-oxidant properties independently of their cholesterol-lowering effect. This study investigated whether atorvastatin prevents aldosterone-induced VSMC inflammation by reducing reactive oxygen species (ROS) production. Vascular smooth muscle cells (VSMC) from WKY rats were treated with 1 μM atorvastatin for 60 min or for 72 h prior to aldosterone (10^−7^ mol/L) stimulation. Atorvastatin inhibited Rac1/2 and p47phox translocation from the cytosol to the membrane, as well as reduced aldosterone-induced ROS production. Atorvastatin also attenuated aldosterone-induced vascular inflammation and macrophage adhesion to VSMC. Similarly EHT1864, a Rac1/2 inhibitor, and tiron, ROS scavenger, reduced macrophage adhesion. Through its inhibitory effects on Rac1/2 activation and ROS production, atorvastatin reduces vascular ROS generation and inhibits VSMC inflammation. Our data suggest that in conditions associated with aldosterone-induced vascular damage, statins may have vasoprotective effects by inhibiting oxidative stress and inflammation.

## 1. Introduction

Aldosterone is a steroid hormone synthesized mainly in the outer layer of the adrenal cortex, the zona glomerulosa [1], although extraadrenal sources of aldosterone have been identified [2]. Aldosterone plays an important role in vascular homeostasis regulating endothelial function, vascular inflammation and remodeling, which are important processes underlying vascular injury in hypertension and atherosclerosis [1,3–5].

Statins, inhibitors of 3-hydroxy-3-methylglutaryl-coenzymeA (HMG-CoA) reductase, revolutionized the treatment of hypercholesterolemia. Statins are competitive inhibitors of HMG-CoA reductase, the rate-limiting enzyme in cholesterol synthesis, thus decreasing endogenous cholesterol synthesis [6,7]. Statins confer cardiovascular protection, which has been confirmed extensively in experimental and clinical studies [8,9]. In addition to lipid-lowering actions of statins, they exhibit a wide array of cardiovascular effects independently of their lipid-reducing properties [10], the so-called pleiotropic effects. Statins influence redox-sensitive processes through putative antioxidant properties and by inhibiting NADPH oxidase (Nox)-derived reactive oxygen species (ROS) generation [10,11]. Recently, we demonstrated that atorvastatin treatment counterbalances type 2 diabetes-associated vascular inflammation and dysfunction and kidney damage, via antioxidant-dependent mechanisms [8,12]. Since vascular cells express functionally active HMG-CoA reductase [13,14], it is also possible that vascular effects of statins may involve local inhibition of this enzyme.

Here, we investigated whether atorvastatin directly modulates aldosterone inflammatory effects in vascular smooth muscle cells (VSCM), and whether this effect is mediated via anti-oxidant properties.

## 2. Material and methods

### 2.1. VSMC culture

The study was approved by the Animal Ethics Committee of the University of Ottawa and performed according to the recommendations of the Canadian Council for Animal Care and in accordance with the Guide for the Care and Use of Laboratory Animals published by the US National Institutes of Health. VSMCs from adult male Wistar-Kyoto rats (16 weeks-old) were euthanized with non-anesthetic gas (carbon dioxide) followed by decapitation. VSMCs derived from mesenteric arteries were isolated and characterized as previously described [15,16]. Low-passage cells (4 to 7) from 3 different batches of cell isolation were studied.

### 2.2. Protocols for cell stimulation

Cells were stimulated with aldosterone (10^−7^ mol/L). Two different times of stimulations were used: 5 and 30 min (min), to assess non-transcriptional effects, or long-term 24 h (h), to assess transcriptional effects. In some experiments cells were pre-exposed for 60 min and 72 h to atorvastatin (10^−7^ mol/L). Cells were also pre-treated for 30 min with EHT1864 (Rac1/2 inhibitor, 10^−6^ mol/L) or tiron (ROS scavenger, 10^−5^ mol/L).

### 2.3. Lucigenin-enhanced chemiluminescence

ROS generation was measured by a luminescence assay with lucigenin as the electron acceptor and NADPH as the substrate, as previously described [15,17].

### 2.4. Western blotting

Total or fractionated proteins from VSMCs were separated by electrophoresis on a polyacrylamide gel, and transferred onto a nitrocellulose membrane. Non-specific binding sites were blocked with 5% skim milk. Membranes were then incubated with specific antibodies overnight at 4 °C described in the cytosol and membrane fractionation. After incubation with secondary antibodies, signals were revealed with chemiluminescence, visualized by autoradiography and quantified densitometrically. Antibody to β-actin (Sigma Aldrich, MO-USA) was used as an internal control.

### 2.5. Cytosol and membrane fractionation

Cytosol to membrane translocation of p47phox, which is essential for NADPH oxidase activation, and Rac1/2 (small G protein necessary for NADPH oxidase activity) was assessed in VSMCs. Cells were lysed and fractionated to obtain cytosol- and membrane-enriched fractions. Western blotting was performed as described using anti-p47phox (Santa Cruz Biotechnology, TX-USA) and anti-Rac1/2 (Cell Signaling, MA-USA). Translocation was determined as the ratio of protein expression in membrane to cytosolic fractions.

### 2.6. Real time RT (reverse transcription)-PCR

Total VSMC mRNA was extracted (Trizol Plus, Invitrogen), purified with chlroform method, and eluted in 20 μL of DEPC-treated water. Complementary DNA was generated by RT-PCR with SuperScript III (Invitrogen). Reverse transcription was performed at 50 °C for 50 min; the enzyme was heat inactivated at 85 °C for 5 min, and real-time quantitative RT-PCR was performed with the SYBR Green Supermix (Bio-Rad Laboratories). The genes analyzed were: Tumor necrosis factor alpha (TNF-α): Fw: ACCACGCTCTTCTGTCTACTG; Rev.: CTTGGTGGT TTGCTACGAC, interleukine 1β (IL-1β): Fw: GCAATGGTCGGGACATA GTT; Rev.: AGACCTGACTTGGCAGAGGA and glyceraldehyde 3-phosphate dehydrogenase (GAPDH): Fw: AAGGTCATCCCAGAGCTGAA; Rev.: GTCCTCAGTGTAGCCCAGGA, which was used as house-keeping gene.

### 2.7. Vascular inflammatory response: macrophage adhesion

Vascular macrophage adhesion was determined according to our previously described methods [16]. Briefly, VSMCs were cultured to confluence in 6-well plates. Growth-arrested VSMCs from WKY rats were stimulated with 10^−7^ mol/L aldosterone for 24 h in the presence or absence of atorvastatin (60 min or 72 h) and inhibitors. Non-stimulated VSMCs served as controls. Rat-derived NR8383 monocyte/macrophage cell lines were obtained from the American Type Culture Collection (Manassas, VA). NR8383 cells, adherent and suspension, were cultured in growth medium (Ham’s F12K with 2 mmol/L _L_-glutamine, 1.5 g/L sodium bicarbonate and 15% heat inactivated fetal bovine serum). For cell fluorescent labeling, macrophages (10^5^ cells/mL) were suspended in 1% bovine serum albumin (BSA)-supplemented phosphate buffered saline containing 1 μmol/L calcein-AM (Molecular Probes, Eugene, OR-USA) and incubated for 20 min at 37 °C. Labeled macrophages were washed twice with phosphate-buffered saline and suspended in Hanks’ buffered salt solution. Fluorescence labeled cells (10^5^ cells/well) were then added to both nonstimulated and stimulated VSMCs layers and were allowed to adhere for 30 min at 37 °C in 5% CO_2_. After the incubation, non-adhered cells were removed by gently washing with pre-warmed Hanks’ buffered salt solution. The number of adherent cells was determined by lysing the cells with 0.1 mol/L NaOH. The cell lysate was transferred to a 96 well plate and the fluorescence intensity was measured with a fluorescence multiwell plate reader (excitation wavelength 485 nm, emission wavelength 535 nm, Cary Eclipse, Varian, CA-USA). VSMC lysate was used as a blank. Experiments were performed in duplicates.

### 2.8. Data analysis

Aldosterone-stimulated effects were determined as the percent increase over control, with the control normalized to 100%. Results are presented as mean ± SEM and compared by one way ANOVA. Values of P < 0.05 were considered to be significant.

## 3. Results

### 3.1. Atorvastatin prevents ROS-generation by inhibiting Rac1/2 and p47phox assembly in VSMCs

**Fig. 1.**
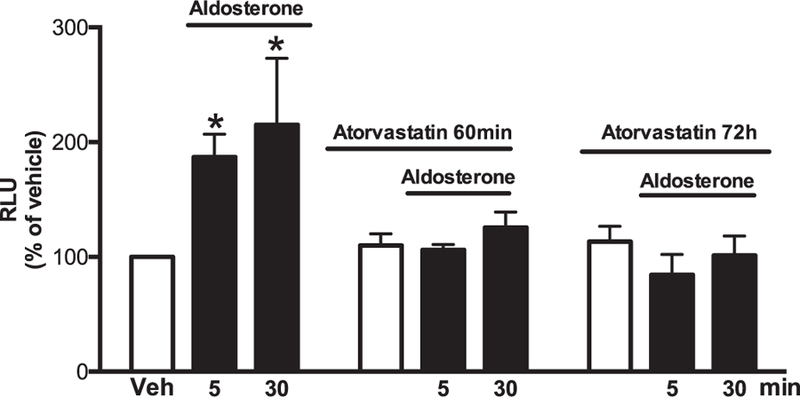
Atorvastatin prevents aldosterone-induced ROS generation in WKY VSMCs. Effects of aldosterone (10^−7^ mol/L) on ROS generation measured by lucigenin chemiluminescence assay in the absence and presence of 10^−7^ mol/L of atorvastatin (60 minutes and 72 hours pre-incubation). Results are mean ± SEM of 5–6 experiments. *P < 0.05, vs. vehicle.

**Fig. 2.**
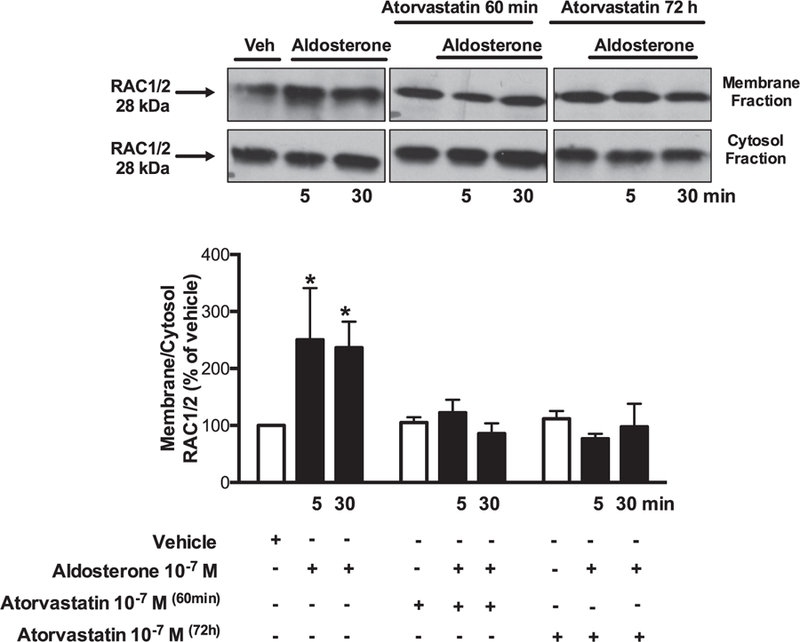
Atorvastatin prevents aldosterone-induced Rac1/2 activity in WKY VSMCs. Effects of aldosterone (10^−7^ mol/L) on Rac1/2 translocation from cytosol to the membrane, in the absence and presence of 10^−7^ mol/L of atorvastatin (60 minutes and 72 hours pre-incubation). Representative immunoblots: Rac1/2 (fractions of membrane and cytosol). Results are mean ± SEM of 5–6 experiments. *P < 0.05, vs. vehicle.

**Fig. 3.**
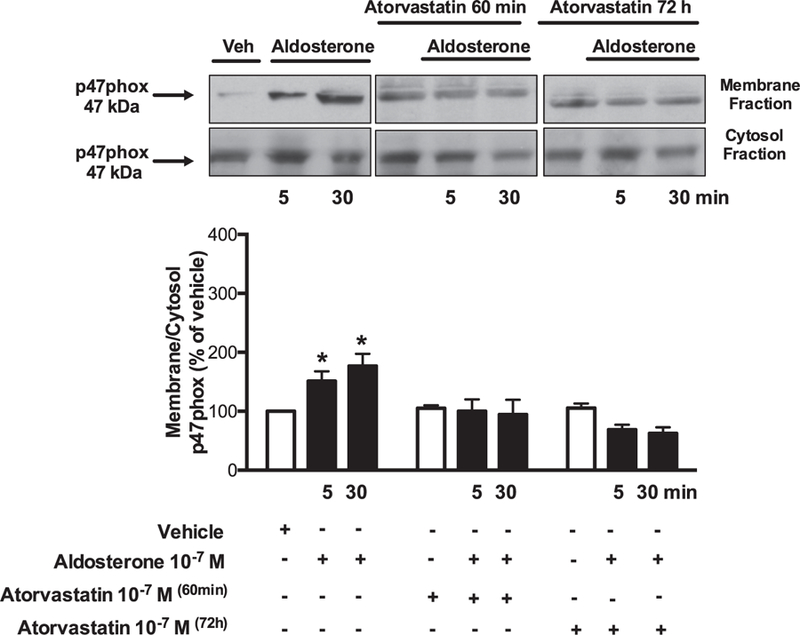
Atorvastatin prevents aldosterone-induced p47pox activity in WKY VSMCs. Effects of aldosterone (10^−7^ mol/L) on p47phox translocations from cytosol to the membrane, in the absence and presence of 10^−7^ mol/L of atorvastatin (60 minutes and 72 hours pre-incubation). Representative immunoblots: p47phox (fractions of membrane and cytosol). Results are mean ± SEM of 5–6 experiments. *P < 0.05, vs. vehicle.

**Fig. 4.**
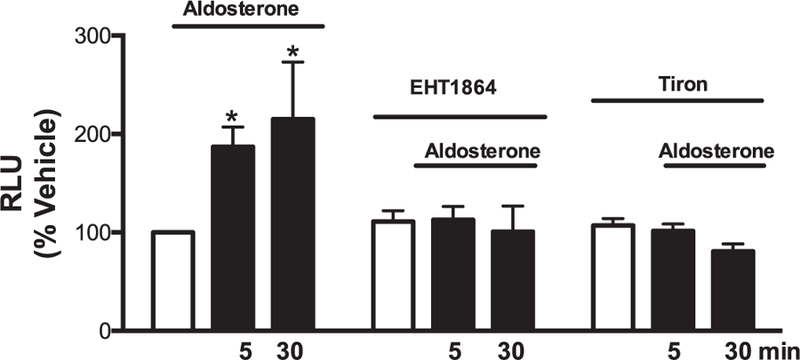
Atorvastatin prevents aldosterone-induced ROS generation via Rac1/2 in WKY VSMCs. Effects of aldosterone (10^−7^ mol/L) on ROS generation measured by lucigenin chemiluminescence assay in the absence and presence of EHT1864 (10^−6^ mol/L) or tiron (10^−5^ mol/L) (30 minute-incubation). Results are mean ± SEM of 5–6 experiments. *P < 0.05, vs. vehicle.

Aldosterone (5 and 30 min) increased ROS production. Atorvastatin pre-incubation (60 min and 72 h) prevented aldosterone-induced ROS generation in VSMCs (Fig. 1). Aldosterone increased Rac1/2 and p47phox translocation from the cytosol to the membrane, which was abrogated by atorvastatin treatment (Figs. 2 and 3). In order to confirm that aldosterone produces ROS via Rac1/2 activation, we incubated VSMC with EHT1864 (Rac1/2 inhibitor), which reduced aldosterone-induced ROS generation, similar to the atorvastatin effects. Tiron (ROS scavenger) also reduced aldosterone-mediated production of ROS (Fig. 4).

### 3.2. Atorvastatin blocks aldosterone-induced vascular inflammation

**Fig. 5.**
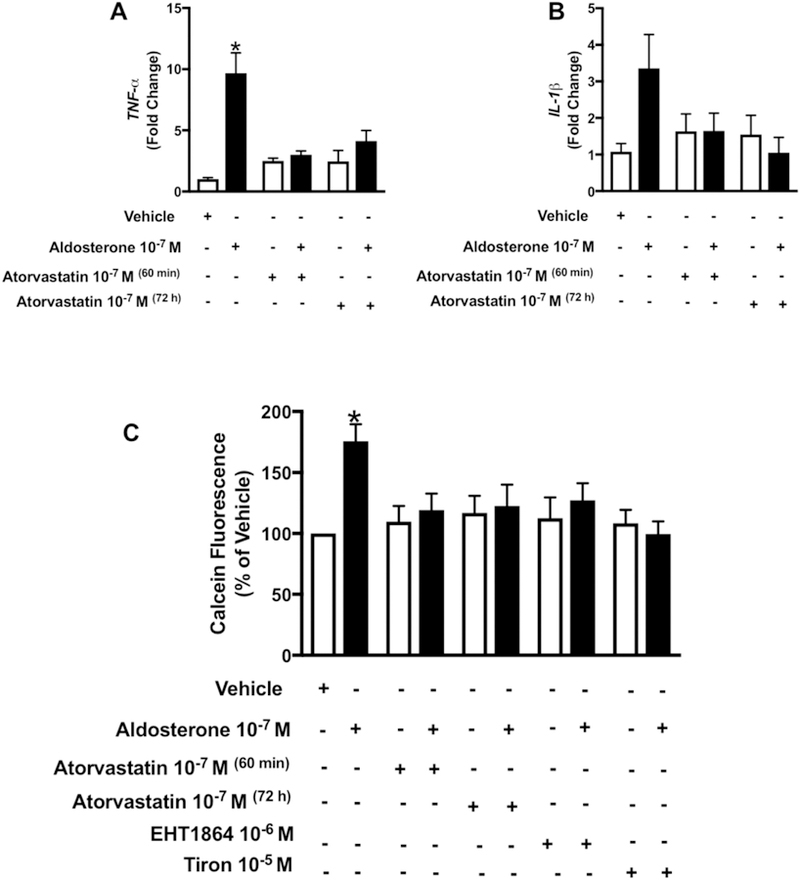
Atorvastatin prevents aldosterone-induced inflammation and macrophages adhesion to WKY VSMCs by Rac1/2 and ROS-sensitive mechanisms. Effects of aldosterone (10^−7^ mol/L) on TNF-α (A) and IL-1β (B) gene expression macrophages adhesion (C) in the absence and presence of 10^−7^ mol/L of atorvastatin (60 minutes or 72 h pre-incubation), EHT1864 (10^−6^ mol/L) or tiron (10^−5^ mol/L) (30 min pre-incubation). Results are mean ± SEM of 4–6 experiments. *P < 0.05, vs. vehicle.

Aldosterone has been associated with vascular inflammation. We analyzed whether statin treatment reduces aldosterone-stimulated macrophage adhesion and inflammatory markers. Aldosterone stimulation significantly increased TNF-α expression and there was a trend to increase IL-1β genes expression. Aldosterone increased the number of adherent macrophages on VSMCs, effects that were attenuated by atorvastatin. Furthermore, Rac1/2 inhibition and ROS scavenging prevented aldosterone-induced vascular inflammatory response (Fig. 5A–C).

## 4. Discussion

Major findings in the present study demonstrate that atorvastatin attenuates inflammatory effects induced by aldosterone in VSMCs by inhibiting Rac1/2 and reducing ROS production. Statins as an adjuvant therapy in the management of cardiovascular diseases such as hypertension and atherosclerosis may have beneficial vascular effects beyond their lipid-lowering effects.

Aldosterone has potent inflammatory and pro-fibrotic actions mediated by mineralocorticoid receptor (MR) activation [16,18,19]. Here, we have shown that aldosterone increases inflammatory markers and macrophage adhesion to VSMCs, supporting a pro-inflammatory vascular phenotype typically observed in atherosclerosis, hypertension and other cardiovascular diseases [21–25]. In the present study, we showed that aldosterone-induced VSMC inflammation is blunted by atorvastatin incubation and that both acute and long-term effects of aldosterone are modulated by atorvastatin. Statins possess broad immunomodulatory and anti-inflammatory properties, e.g. in endothelial cells statins increase endothelial nitric oxide synthase (eNOS) mRNA expression and nitric oxide bioavailability, decreases chemokines receptors, adhesion protein and cytokines production [20]. In addition, statin treatment reduces vascular remodeling and oxidative stress in angiotensin II treated mice, as well as attenuates type I collagen formation in isolated VSMC under angiotensin II stimulus via ROS production [21].

Besides being considered a marker for inflammation, ROS have been considered as a seconder messenger for the inflammatory response [4,15,19,22–24]. The deleterious effects produced by aldosterone have been mainly associated with ROS production [3,4,25,26]. Here, we show that aldosterone elevates ROS production in VSMC, which is abrogated by atorvastatin. Small GTPases such as Rac1/2 are essential for NADPH oxidase activation. Our findings suggest that atorvastatin might reduce aldosterone-induced ROS generation by inhibiting Rac1/2 and consequently Nox-activation, since atorvastatin pre-incubation blocked aldosterone-induced Rac1/2 and p47phox activation. Nox1 and Nox2 are constitutively associated with p22phox, and the full activation of these complexes requires interaction with other cytosolic subunits, including p47phox [8,23,27]. Further supporting a role for Rac1/2, NADPH oxidase and ROS in aldosterone-induced inflammation are the findings that tiron, the ROS scavenger, and EHT1864, Rac1/2 inhibitor, prevented macrophage adhesion to VSMC induced by aldosterone. This was associated with reduced ROS production and suggests that atorvastatin reduces VSMCs inflammation by inhibiting Rac1/2 and reducing ROS production, possibly mediated by Nox enzymes.

Statins are considered potent inhibitors of cholesterol biosynthesis. However, the overall benefits observed with statins also include effects beyond cholesterol lowering effects, e.g.: blocking Small GTPase such as Rac1/2 [10,13,20,21,28]. Here, 60 min of pre-exposure to atorvastatin generated vascular beneficial effects most likely by directly inhibiting Rac1/2 activity, whereas 72 h of pre-treatment might produce beneficial effects via dual actions: by lowering cholesterol content and by inhibiting Rac1/2 activity. The cell membrane contains lipid rafts, which have high concentrations of cholesterol and sphingolipid and which are responsible for stabilization of several proteins including the NADPH oxidases [29,30]. Although we did not check the lipid rafts content in the present study, 72 h of statin incubation might be inhibiting ROS production and vascular inflammation by disrupting lipid rafts content. Some studies have shown that statin long-term incubation reduces the lipid rafts content [31,32].

We have not analyzed the effects of statin treatment in experiments in vivo in the present study, but it is worth mentioning that statins can reduce aldosterone plasma levels in hypertensive and diabetic patients, as well as diminish Ang II-induced aldosterone secretion in mouse zona glomerulosa cells [33]. These findings reinforce the notion that atorvastatin may protect the vasculature against pro-inflammatory effects of aldosterone, as well as reduce aldosterone production.

In conclusion, our findings indicate that atorvastatin prevents aldosterone-induced vascular inflammation associated with Nox–mediated ROS generation. Our findings indicate that atorvastatin may protect the vasculature in diseases that are associated with elevated levels of aldosterone. In addition, we propose that statins might have dual beneficial effects in cardiovascular disease through its lipid-lowering actions and direct pleiotropic vascular effects.
